# Selection of Earmuffs and Other Personal Protective Equipment Used in Combination

**DOI:** 10.3390/ijerph16091477

**Published:** 2019-04-26

**Authors:** Emil Kozlowski, Rafal Mlynski

**Affiliations:** Department of Vibroacoustic Hazards, Central Institute for Labour Protection-National Research Institute, 00-701 Warsaw, Poland; rmlynski@ciop.pl

**Keywords:** hearing protection, hearing protectors, earmuffs, personal protective equipment, sound pressure level, noise

## Abstract

In a work environment, in addition to noise, people may be exposed to other harmful factors. Therefore, they wear both hearing protectors and other personal protective equipment (OPPE). Incorrect use of such a combination may increase the risk of hearing loss. The aim of this study was to determine whether the simultaneous use of earmuffs and other personal protective equipment could affect the effectiveness of hearing protection. The study was carried out under laboratory conditions using an acoustic test fixture. This fixture replicated the anatomical shapes of the head and the pinnae, and was also equipped with ear simulators. The study was carried out for five models of earmuffs and eight models of other personal protection equipment. We found that a change in the sound pressure level (SPL) under the earmuffs when using a full face respirator could reach up to 40 dB. On the other hand, the use of a half respirator had practically no adverse impact on the efficiency of hearing protection. In the selection process, it is recommended to consider safety spectacles equipped with thin temples, and half respirators equipped with band adjustment elements positioned on the facial part, rather than the back, of the user’s head.

## 1. Introduction

The reduction of exposure to noise in the workplace is possible by means of engineering measures, for example, by introducing acoustic treatments, soundproof booths, and administrative methods. These include rotating employees between workplaces with different noise conditions or limiting the time of exposure to noise. However, there are situations where the only method of reducing exposure of workers to noise is the use of hearing protectors. Only properly selected and correctly used hearing protectors can provide full hearing protection. The selection of hearing protectors consists in calculating the A-weighted sound level under them based on the values of noise parameters present in the workplace and sound attenuation provided by the hearing protectors [1]. The sound attenuation of hearing protectors is specified by manufacturers in the user manual. Quite often, there is a difference between the assumed effectiveness of hearing protection resulting from the use of hearing protectors based on the sound attenuation values depicted in the user manual and the effectiveness of protection occurring under real conditions [2,3,4,5,6,7,8,9,10]. The reason for these differences lies in the fact that the tests conducted to determine sound attenuation of hearing protectors are performed under controlled laboratory conditions, usually by a group of trained subjects, on brand new samples of hearing protectors [11,12,13]. Meanwhile, the use of hearing protectors under real conditions often differs from conditions in the laboratory. For example, workers often do not pay due attention to the correct placement of hearing protectors [14,15] (this applies mainly to earplugs). They also use old, worn-out hearing protectors, whose effectiveness is limited [16,17,18]. In working establishments, there are often situations in which workers are exposed to harmful factors other than noise and therefore they simultaneously wear earmuffs and other personal protective equipment (OPPE). This combination may result in a decrease in the effectiveness of hearing protection due to the appearance of gaps in the cushions of the earmuffs. Research on this issue is limited and has only been conducted on a small number of samples. Abel et al. [19] compared the sound attenuation of one model of earmuffs attached to a protective helmet used without OPPE and worn simultaneously with safety spectacles and an air-purifying respirator. Their research showed that as a result of using OPPE, earmuff attenuation deteriorated by up to 9 dB. A similar study, also using a single model of earmuffs, was carried out by Chung et al. [20]. The tests were related to the impact of using glasses and caps and having long hair on earmuff attenuation. They indicated that the greatest reduction in earmuff attenuation, by more than 10 dB, was due to the presence of a cap. When earmuffs were used by persons also using corrective glasses or having long hair, earmuff attenuation was lower by approximately 5 dB. Testing of hearing protection efficiency in the case of earmuffs and OPPE in combination, carried out on a much larger group of personal protective devices, was completed by Lemstad and Kluge [21] and Wells et al. [22]. In the case of the first of these works [21], the use of safety spectacles with earmuffs resulted in the reduction in earmuff attenuation by 14 dB in the lower frequency range. With respect to Wells et al. [22], they showed that safety spectacles could lower the noise reduction rating (NRR) parameter in earmuffs by 11 dB. The works discussed above concern the simultaneous use of earmuffs and OPPE. It should be noted that it is also possible to use earplugs simultaneously with OPPE. Then, because the user inserts the earplugs into the ear canal, there will be no effect of OPPE on the effectiveness of hearing protection. In addition to the discussed situations regarding passive hearing protectors, the combination of level-dependent earmuffs and safety spectacles is often used in the presence of impulse noise. Despite the presence of electronic circuits, the impact of OPPE on the level-dependent earmuffs should be the same as in the case of passive earmuffs. This is due to the fact that in the presence of high-level noise, electronic circuits do not transmit sound under the earmuffs [1].

The purpose of this study was to determine how the simultaneous use of earmuffs and OPPE could affect the effectiveness of hearing protection. Unlike previous studies [18,19,20,21,22], the present work used an acoustic test fixture, designed specifically to assess the acoustic properties of hearing protectors [23]. In addition, the tests included a large set of both earmuffs and other personal protective equipment.

## 2. Materials and Methods

### 2.1. Earmuffs

**Figure 1:**
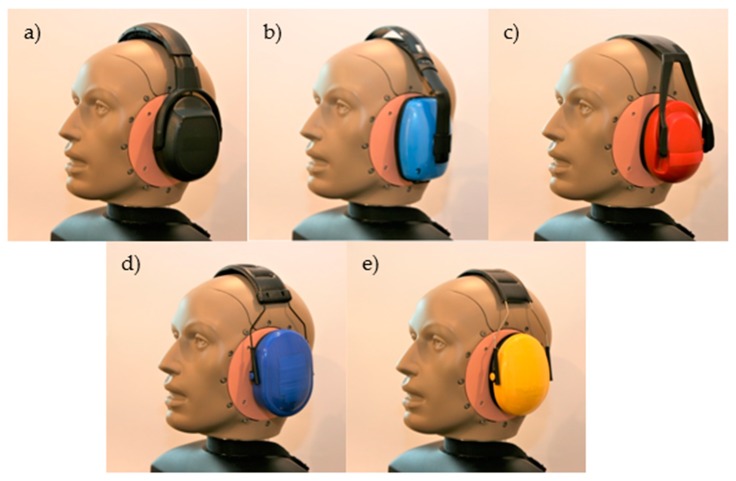
Tested earmuffs (EM): (**a**) EM1, (**b**) EM2, (**c**) EM3, (**d**) EM4, and (**e**) EM5.

The research was carried out for five popular models of earmuffs used in the industry (marked for the purpose of this work as EM1, EM2, EM3, EM4, and EM5). The tested earmuffs are presented in Figure 1. These earmuffs differ in the nominal sound attenuation (shown in the user manual), design of the headband (plastic or metal, mounted on the side or top of the cup), as well as structure of the cups (shape, dimensions, mass) and the cushions (contact surface area). 

### 2.2. Other Personal Protective Equipment

**Figure 2:**
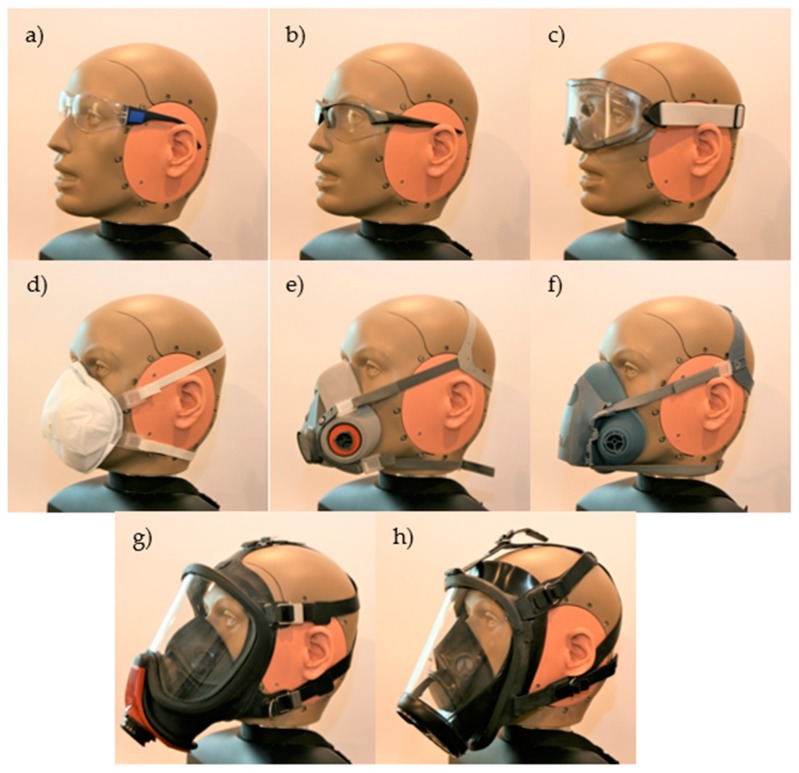
Tested other personal protective equipment (OPPE): (**a**) safety spectacles SS1, (**b**) safety spectacles SS2, (**c**) goggles G, (**d**) disposable respirator DR, (**e**) reusable half respirator RHR1, (**f**) reusable half respirator RHR2, (**g**) reusable full face respirator RFFR1, and (**h**) reusable full face respirator RFFR2.

The following other personal protective equipment (OPPE) was selected for testing with the above-specified earmuffs: two models of safety spectacles (marked for the purpose of this work as SS1 and SS2), one model of goggles (marked as G), one model of disposable respirator (marked as DR), two models of reusable half respirators (marked as RHR1 and RHR2), and two models of reusable full face respirators (marked as RFFR1 and RFFR2). The selected spectacles differed in temple dimensions, whereas the selected respirators differed in the design of the facial elements and the type of headbands used for adjustment. The tested OPPE is presented in Figure 2.

### 2.3. Measuring Setup

**Figure 3:**
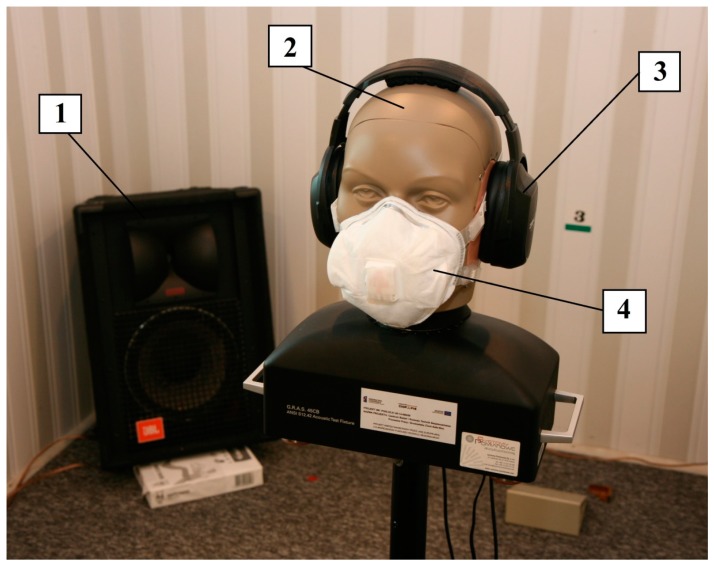
Measuring setup used for measurements of sound pressure level (SPL) under the earmuffs worn in combination with OPPE: 1—one of the four speaker sets, 2—acoustic test fixture, 3—tested earmuffs, 4—example of OPPE.

The tests consisted of measuring the sound pressure level (SPL) in 1/3 octave bands (for center frequency within the range from 100 Hz to 10 kHz) and an A-weighted equivalent SPL (L_Aeq_) under the earmuffs. The tests were conducted in a diffuse field chamber equipped with four JBL 4208 loudspeaker sets (HARMAN International, Stamford, CT, USA). The test signal was broad-band noise (L_Aeq_ = 106.5 dB). The noise was fed to the loudspeaker sets via a path comprising a Sony CDP-XE270 CD player (Sony Corporation, Tokyo, Japan), 2 Crown Macro-Tech 2400 power amplifiers (HARMAN International, Stamford, CT, USA), a JBL DCS260 limiter (HARMAN International, Stamford, CT, USA) and a Yamaha YDG2030 equalizer (YAMAHA Corporation, Shizuoka, Japan). Measurements of the SPL under the earmuffs were performed using two GRAS 40BP microphones (GRAS Sound & Vibration A/S, Holte, Denmark) placed in both ear simulators of the GRAS 45CB acoustic test fixture (GRAS Sound & Vibration A/S, Holte, Denmark). The measurements were carried out in two situations: with the earmuffs put on together with one device from among the above-mentioned OPPE and with only earmuffs put on without any OPPE. The end result of the study was the difference between the SPLs obtained in both of those situations. This difference determines how the SPL changes under the earmuffs in the case of using OPPE, which consequently also affects the effectiveness of the hearing protection of an employee applying personal protective equipment. The test for each model of earmuffs was repeated three times; therefore, the final result was obtained on the basis of six measurements (three measurements per each of the earmuff cups). The acoustic test fixture used in the tests meets the requirements of the ANSI/ASA S12.42 [23] standard on the measurement of insertion loss of hearing protectors, intended for testing in the presence of continuous and impulsive noise. This test fixture is characterized by the fact that it replicates the anatomical shapes of the human head and the pinna. It is also equipped with ear simulators shaping the frequency characteristics of the fixture to reflect the characteristics observed in people. In addition, in order to reproduce the conditions of using hearing protectors, the fixture has a preheating system maintaining the surface temperature of the test fixture that remains in contact with the elements of the protection devices at the level of 37 °C. To reproduce human skin properties, this surface is made of silicone rubber with a hardness of 55 (Shore 00). With these solutions, the test fixture used in the study is not a device designed solely to control product quality, but it also acts as a specialist device used to test the acoustic properties of hearing protectors. The data acquisition path used a Brüel & Kjær 3052-A-030 measurement unit (Brüel & Kjær, Nærum, Denmark) and the GRAS 12AA power module (GRAS Sound & Vibration A/S, Holte, Denmark), supplying microphones and amplifying the measurement signal. The measuring setup used for measurements of the SPL under the earmuffs used in combination with OPPE is presented in Figure 3.

### 2.4. Statistical Analysis

To determine whether the use of OPPE affected the SPL under the earmuffs, statistical analysis was performed using the non-parametric Wilcoxon signed-rank test. The calculations were performed using MATLAB R2010b version 7.11.0.584 (MathWorks Inc., Natick, MA, USA).

## 3. Results

**Figure 4:**
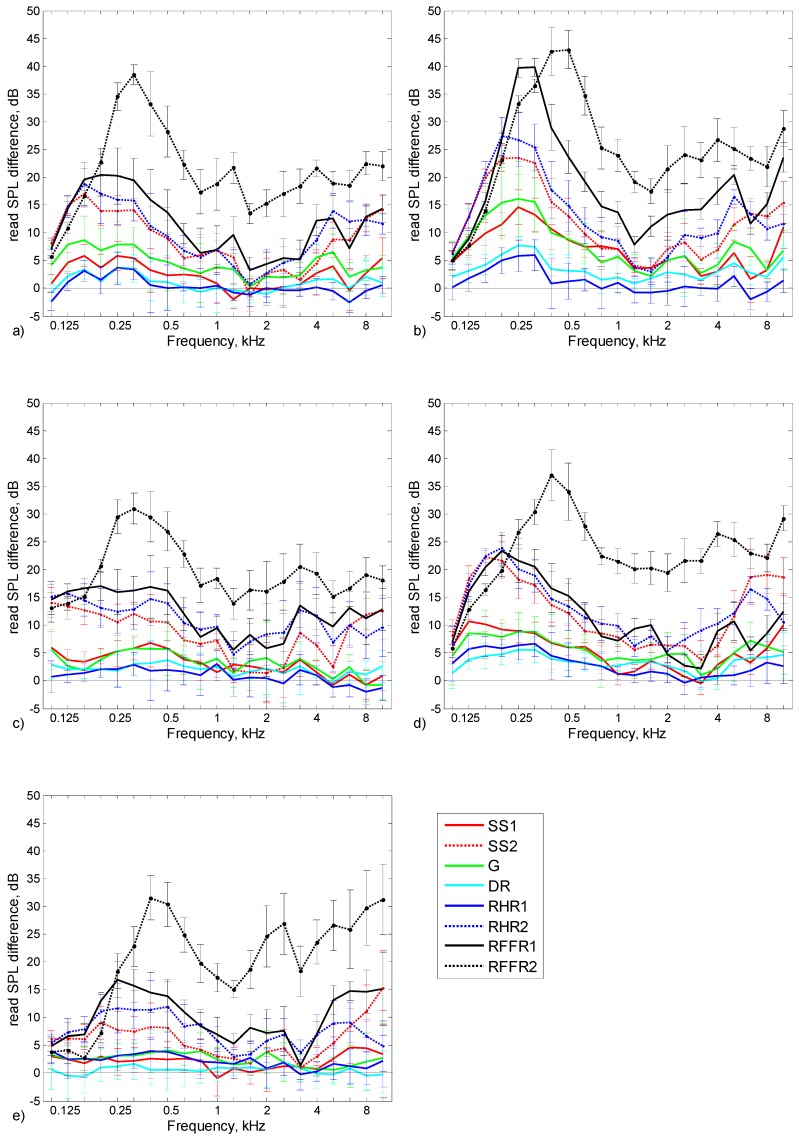
Difference between SPLs in 1/3 octave bands under earmuffs used in combination with OPPE and without OPPE: (**a**) EM1; (**b**) EM2; (**c**) EM3; (**d**) EM4; and (**e**) EM5. SS—safety spectacles, G—goggles, DR—disposable respirator, RHR—reusable half respirators, RFFR—reusable full face respirators. Error bars show standard deviations.

Figure 4a presents the changes in SPLs in 1/3 octave bands under the EM1 earmuffs with the assumption of other personal protective equipment (OPPE) being used. The changes in the SPL presented in Figure 4a refer to eight situations where the EM1 earmuffs were put on in turn with safety spectacles SS1 and SS2, goggles G, disposable respirator DR, reusable half respirators RHR1 and RHR2, and with reusable full face respirators RFFR1 and RFFR2. Likewise, Figure 4b–d,f presents changes in the SPL determined under the cups of earmuffs EM2, EM3, EM4, and EM5, respectively.

Table 1 presents the L_Aeq_ values determined under the earmuffs using an acoustic test fixture in a situation where the earmuffs were put on without OPPE and worn simultaneously with safety spectacles SS1 and SS2, goggles G, disposable respirator DR, reusable half respirators RHR1 and RHR2, and with reusable full face respirators RFFR1 and RFFR2.

The results of the studies shown in Figure 4 and in Table 1 indicate that from among the tested OPPE, full face respirators have the greatest impact on the SPL under the earmuffs. For all earmuff models, the RFFR2 respirators used in combination with them caused the SPL values obtained under the earmuffs, taking into account the entire considered frequency range (100 Hz–10 kHz), to undergo a statistically significant change (*p* < 0.01). Table 2 presents all *p*-values obtained in the analysis of whether the SPL in the 1/3 octave bands determined under the earmuffs without OPPE, and the SPL designated under the earmuffs worn simultaneously with OPPE, had a statistically significant difference.

When wearing the RFFR2 respirator, the SPL under the earmuffs increased over the entire frequency range, especially within the range of 250–500 Hz. The largest increase in the SPL under the influence of the RFFR2 respirator, reaching up to 40 dB, was present for the EM2 earmuffs. This increase in the SPL means that, as a consequence, attenuation of the earmuffs at frequencies of 250 and 500 Hz is therefore reduced by 40 dB. The impact of the RFFR2 respirator was so significant that L_Aeq_ under the earmuffs was lower by 9–16 dB in the case of using earmuffs in combination with a respirator relative to when the earmuffs were not used at all (L_Aeq_ = 106.5 dB). This proves the potentially very low protection of the hearing organ should the earmuffs be used simultaneously with the RFFR2 respirator. Less impact on the SPL under the earmuffs was observed with the RFFR1 respirator. Using this device caused the SPL in the 1/3 octave bands to increase to a maximum of approximately 15–25 dB in the case of earmuffs EM1, EM3, EM4, and EM5. In the case of the EM2 earmuffs, a greater impact of RFFR1 on the SPL under the earmuffs was observed. The data presented in Figure 4b show that both types of full face respirators attained similar values in the low frequency range, whereas in the case of the RFFR1 respirator, the maximum was slightly shifted towards the lower frequencies. For medium and high frequencies, as was the case with other earmuffs, the impact of the RFFR1 respirator on the SPL under the EM2 earmuffs was less than for the RFFR2 respirator. The use of the RFFR1 respirator caused the L_Aeq_ value under the earmuffs to increase by approximately 10–20 dB (Table 1), depending on the earmuff model, and thus reduced the effectiveness of hearing protection.

In principle, the use of half respirators with earmuffs should cause smaller changes to the SPL than full face respirators. This is due to the structure of these devices. This was the case with the RHR1 respirator. Depending on the model of the earmuffs, the SPL in 1/3 octave bands changed mainly within the range of low frequencies by 3–7 dB. However, statistical analysis demonstrated that despite changes in the SPL observed in a certain range, when taking into account the entire considered frequency range, those changes will not be significant in the case of all considered earmuffs (*p* = 0.35 ÷ 1). This was not the case with the RHR2 respirator. The use of this respirator in combination with earmuffs resulted in statistically significant (*p* < 0.035) changes in the SPL under the earmuffs. The SPL increased from approximately 10 to 25 dB and the largest changes again affected the EM2 earmuffs. It should also be noted that despite the larger size of the RFFR1 respirator, the impact of its use on the SPL under the earmuffs was similar in most cases to the impact of the RHR2 respirator. In both situations, changes in the SPL in the 1/3 octave bands (Figure 4) and in L_Aeq_ (Table 1) were similar. The EM2 earmuffs were an exception, where the impact of the RFFR1 respirator was greater in this case. 

As with the RHR1 respirator, wearing a disposable respirator (DR) had no statistically significant effect on the SPL in the 1/3 octave bands under the earmuffs (*p* = 0.07 ÷ 0.86). In addition, the values of L_Aeq_ (Table 1) measured without and with the DR respirator were similar.

As in the case of earmuffs used in combination with respiratory protective equipment, the impact of the use of personal protective equipment for the eyes and the face (safety spectacles and goggles) on the effectiveness of hearing protection varied. In the case of four models of earmuffs, the simultaneous use of SS2 spectacles resulted in a statistically significant increase in the SPL in the 1/3 octave bands. The greatest changes in the SPL, exceeding 20 dB at frequencies of 160 and 200 Hz, were observed for earmuffs EM2 and EM4. Smaller changes in the SPL in the 1/3 octave bands occurring while using the SS2 spectacles, which were not statistically significant (*p* = 0.11), were observed in the case of the EM5 earmuffs. Unlike the case of the SS2 spectacles, the use of the SS1 spectacles did not statistically impact the SPL under the four earmuff models. Only the use of the SS1 spectacles simultaneously with the EM2 earmuffs caused the SPL in the 1/3 octave frequency bands under those earmuffs to increase to the point of statistical significance (*p* < 0.01). Similar test results were achieved in the case of the simultaneous use of earmuffs and goggles (G). Additionally, for four out of five earmuff types, the effect of using the G goggles on the SPL in the 1/3 octave bands was not statistically significant. 

When analyzing the data in Table 1, it can be noted that the use of the SS1 spectacles resulted in the value of L_Aeq_ under the earmuffs becoming higher by approximately 1–6 dB. However, in the case of the SS2 spectacles, the value of L_Aeq_ increased by approximately 6–13 dB.

**Figure 5:**
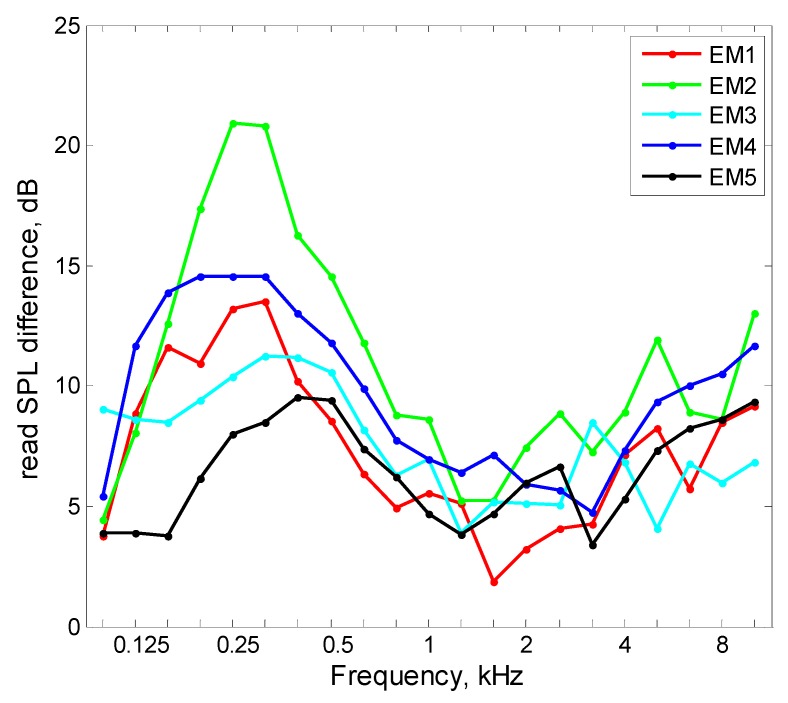
Average difference between the SPLs in the 1/3 octave bands measured under the earmuffs (EM) used in combination with OPPE (average of eight tested OPPE) and without OPPE.

Figure 5 shows how different models of earmuffs are susceptible to changes in the SPL in the 1/3 octave bands resulting from the use of OPPE. The diagrams in Figure 5 present the average values of changes in the SPL under the earmuffs, taking into account all tested OPPE. 

By comparing the values shown in the diagrams in Figure 5, it can be concluded that for most frequency bands the change in the SPL under the earmuffs induced by the use of OPPE was similar, and its span was approximately 5 dB. The case was different in the frequency range of 125–500 Hz, for which a noticeably greater effect of OPPE on the SPL was visible under the EM2 earmuffs in relation to other earmuffs. However, in the case of the EM5 earmuffs, the situation was quite the opposite, that is, the use of OPPE had the smallest impact on those earmuffs. The values of the changes in the SPL in the case of the EM2 earmuffs were greater by 13 dB than the EM5 earmuffs. In addition to the presented mean values, the standard deviation values are also shown in Figure 4. Standard deviation values were calculated based on the values of standard deviations determined in the SPL measurement under the earmuffs and under the earmuffs used with OPPE. It should be noted that the highest standard deviation values of the SPL measurement under the earmuffs in this study (2.7–5.0 dB) are comparable or less than the standard deviation values of the sound attenuation measured using the real ear attenuation at threshold (REAT) method (3.1–6.1 dB). Based on this, it can be concluded that the inaccuracy of measurements performed in this work is similar to the inaccuracy of the measurement of sound attenuation commonly used for testing hearing protectors.

## 4. Discussion

The use of the acoustic test fixture in this study allowed for less time-consuming determination of the impact of OPPE on the effectiveness of hearing protection from earmuffs compared to the subjective real ear attenuation at threshold (REAT) method used by default for testing hearing protectors. Because the applied method is based on the use of a measuring microphone, the SPL could be determined in all 1/3 octave frequency bands within the range of 100 Hz–10 kHz as opposed to the REAT method [11,12,13], where the properties of the hearing protection devices are determined for a limited number of bands based on the hearing threshold of people. The use of the acoustic test fixture allowed for a more accurate analysis of the SPL under the earmuffs. Moreover, due to the fact that attenuation values were not measured but that the changes in attenuation associated with the use of OPPE were obtained, the share of people in the study could be replaced by the use of the acoustic test fixture. 

The acoustic test fixture GRAS 45CB used in this study is a fairly new solution and so far, it has been used mainly for testing hearing protectors under the conditions of impulsive noise [24,25]. The microphone in real ear (MIRE) [20,21,26,27] method and the above-mentioned REAT method [19,22] have been used more often for testing the impact of OPPE on earmuff attenuation. The MIRE method consists in measurements of the SPL (e.g., under hearing protectors using a miniature microphone placed in the external ear canal or in the person’s pinna). For example, the MIRE method was used by Lemstad and Kluge [21] to analyze the impact of the use of safety spectacles on earmuff attenuation. That work showed that earmuff attenuation may be smaller by as much as 14 dB when used in combination with safety spectacles with thick temples. However, when spectacles with thinner temples were used, the decrease in earmuff attenuation was up to 4 or 5 dB. Similar observations (i.e., the impact of the type of temples in safety spectacles used in combination with the earmuffs on the effectiveness of hearing protection is large) can also be made on the basis of the results of this work. In the case of the SS1 spectacles, which were equipped with thin temples, the change in the SPL under the earmuffs was distinctly smaller than that of the SS2 spectacles equipped with wide temples. In addition, despite the fact that both studies focused on different models of earmuffs and different models of spectacles, the changes in the SPL in the case of three earmuffs, namely, EM1, EM3, and EM5, were similar to what was presented in the work by Lemstad and Kluge [21]. When analyzing the effect of spectacles on earmuff attenuation as a function of frequency, the greatest changes in attenuation presented in the work of Lemstad and Kluge [21] were observed within the range of 160–200 Hz, which is also similar to the results presented in the current study, where the changes in the SPL occurred mainly within the low frequency range. The MIRE method was also used by Mlynski and Kozlowski [26] to test earmuff attenuation used in combination with spectacles. Apart from the MIRE method, as opposed to this study, the authors analyzed a change in earmuff attenuation in the presence of impulsive noise. Despite another test signal (a shot from a starter pistol) in the case of their study [26], they also observed a significant impact of the size of the spectacles’ temples on the obtained attenuation of impulsive noise. A change of spectacles equipped with wide temples for those with thin temples increased earmuff attenuation by as much as 13 dB. Wells et al. [22] also tested the impact of using safety spectacles on earmuff attenuation, whereas, as opposed to the above-described studies, the subjective REAT method was used for that purpose. That work also demonstrated that the size of the spectacles’ temples had an impact on the attenuation of earmuffs used in combination with the spectacles. In the case of spectacles equipped with thin temples, the NRR parameter of the earmuffs decreased by 2 dB, and it dropped by 9 dB in the case of wide temples. Moreover, it was found, which was not a part of the work, that gel-filled cushions tend to deform less when using spectacles than cushions filled with foam. This results in greater earmuff attenuation for the simultaneous use of spectacles and earmuffs equipped with gel-filled cushions compared with cushions filled with foam. Abel et al. [19] also applied the REAT method to determine the attenuation of earmuffs used simultaneously with other personal protective equipment. Tests with safety spectacles indicated a slight reduction up to 5 dB in earmuff attenuation. Those results are therefore similar to those presented in the present work in the case of SS1 spectacles with thin temples. Abel et al. [19], apart from the impact of safety spectacles, also studied the impact of half respirators on earmuff attenuation. Their tests related to only one model of respirator and, like the spectacles, the half respirator caused a reduction in earmuff attenuation by a maximum of 5 dB. A similar result (i.e., an increase in the SPL under the earmuffs by approximately 5 dB) was obtained in the present work when the RHR1 half respirator was used. As was the case with safety spectacles, there were large variations between changes in the SPL in the case of half respirators, depending on the model of the equipment used. This is because half respirators may be equipped with different types of headbands. For example, the RHR2 respirator is equipped with an element for adjustment of the headbands at the back of the head (Figure 2f), unlike the RHR1 half respirator, where this element is located on the facial part of the half respirator (Figure 2e). The adjustment element located at the back of the head causes the earmuffs to protrude from the user’s head, which in turn results in a significant increase in the SPL under the earmuffs. A similar situation occurs with full face respirators. In the case of one of the tested full face respirators (RFFR2), significant changes in the SPL under the earmuffs were observed over the entire frequency range. The reason for such large changes is that these masks have headbands equipped with convex adjustment elements (Figure 2h), thus creating gaps between the cushions of the earmuffs and the user’s face. On the other hand, the RFFR1 respirator has less effect on the change in the SPL under the earmuffs because it is equipped with flat metal elements for adjusting the headbands (Figure 2g). The above observations relating to this work were also made in the previous work of Kozlowski and Mlynski [27]. That work relates to studies on the impact of respiratory protective equipment on earmuff attenuation but, unlike this work, the authors applied the MIRE method. When comparing the test results obtained with the acoustic test fixture and the miniature microphone, it can be observed that in the case of a test fixture, the change in the SPL under the earmuffs is higher than for measurements with the MIRE method. In the case of the full face respirator and the MIRE method, the changes reach up to 20 dB and they are at 15 dB in the case of half respirators. The largest changes observed in the present study took place for full face respirators and half respirators, that is, they reached up to 40 and 25 dB, respectively. Despite those differences, the very nature of changes in the SPL is similar in both studies, that is, the greatest impact of OPPE was observed in the low frequency range. It should be added that each method is based on different assumptions. The differences between the methods are related to the impact of individuals’ features in the test group or they result from the use of the device in an average manner, reflecting features of those persons. Irrespective of the differences between the method using the acoustic test fixture and the MIRE method, both allow for selecting sets of personal protective equipment least susceptible to the impact of combined use of earmuffs and OPPE. However, the method using the test fixture allows researchers to obtain the results more quickly and without the involvement of a group of people for testing.

## 5. Conclusions

Studies have shown that the simultaneous use of earmuffs and OPPE affect the SPL under the earmuffs, most often causing it to increase. This increase consequently reduces the effectiveness of the hearing protection. 

The design of OPPE used simultaneously with the earmuffs affects the SPL values achieved. In the case of half respirators, there was a significant difference in their impact on the SPL under the earmuffs due to the different positioning of the headband adjustment element. In a situation where the half respirator had the adjustment element located on the facial part, its impact on the SPL was practically negligible. However, when the adjustment element was located at the back of the head, the effect of the half respirator on the SPL was significant. 

A similar conclusion can be derived from studies with safety spectacles. Safety spectacles with thin temples impact the effectiveness of hearing protection of the earmuffs to a much lesser extent than those with wider temples.

When comparing the changes in the SPL for various models of earmuffs caused by the use of OPPE, it can be observed that these changes are different for each model. In other words, the individual earmuff models of are more or less susceptible to the influence of OPPE. This is likely due to the design of the cushions, which in different models of earmuffs creates smaller or larger gaps between the cushions and the head skin as a result of using OPPE.

These observations lead to the conclusion that when it is necessary to use the earmuffs and OPPE in combination, the negative impact of OPPE on the effectiveness of hearing protection can be reduced and, in some cases, eliminated by the appropriate choice of OPPE together with the proper choice of earmuffs.

## Figures and Tables

**Table 1:** L_Aeq_ values determined under earmuffs used without OPPE and in combination with OPPE. EM—earmuffs, SS—safety spectacles, G—goggles, DR—disposable respirator, RHR—reusable half respirators, RFFR—reusable full face respirators.

Earmuffs	OPPE								
	Without	SS1	SS2	G	DR	RHR1	RHR2	RFFR1	RFFR2
EM1	71.1	72.5	78.8	74.6	71.4	71.1	80.2	81.5	94.2
EM2	71.1	77.0	82.6	78	74.4	71.9	83.8	91.2	97.9
EM3	69.2	72.1	76.9	72.6	71	69.6	79.7	80.8	90.7
EM4	69.8	74.5	83.2	75.1	72.6	72.1	82.9	82.1	94.8
EM5	73.7	75.5	79.5	76.1	74.3	75.9	81.9	84.4	95.3

**Table 2:** *p*-values determined for comparison of SPLs under the earmuffs used without OPPE and with OPPE. EM—earmuffs, SS—safety spectacles, G—goggles, DR—disposable respirator, RHR—reusable half respirators, RFFR—reusable full face respirators. *p*-values marked in bold indicate statistically significant changes.

Earmuffs	*p*-Value							
	Without SS1	Without SS2	Without G	Without DR	Without RHR1	Without RHR2	Without RFFR1	Without RFFR2
EM1	0.42	**0.03**	0.15	0.86	1	**<0.01**	**<0.01**	**<0.01**
EM2	**<0.01**	**<0.01**	**<0.01**	0.07	0.71	**<0.01**	**<0.01**	**<0.01**
EM3	0.37	**0.02**	0.33	0.41	0.74	**<0.01**	**<0.01**	**<0.01**
EM4	0.14	**<0.01**	0.07	0.26	0.35	**<0.01**	**<0.01**	**<0.01**
EM5	0.41	0.11	0.41	0.84	0.51	**0.03**	**0.01**	**<0.01**
